# Ca'-ing Canny Throughout the Nation?

**Published:** 1919-09-13

**Authors:** 


The Hospital

The Workers' Newspaper of Administrative Medicine and Institutional Life, Administration, National Insurance and Health.

No. 1736, Vol. LXVI. SATURDAY, SEPTEMBER 13, 1919. PRICE TWOPENCE

### CA'-ING CANNY THROUGHOUT THE NATION?

The Premier's speech at the rising of Parliament contained an important statement against the practice of "ca'ing canny." He told us of lathes being run without doing work, the deliberate reduction of the output of coal, and like signs of slackness among the working people. But Mr. Lloyd George went further, and said he did not think this evil is confined to labour, for he had had some evidence of it amongst employers and managers. This is an important point, because it shows that this practice may be present among the educated classes. However annoyed we may feel with the working man who believes that if he does less work himself there will be more for his mates to do, we cannot seriously blame him for what is a mistaken loyalty to his pals; we can only try to educate him up to a better understanding and to urge him to work always with a will until he gains such understanding. If, however, we find the same practice extending to other classes, we can feel nothing but indignation at such behaviour. Such men are supposed to be educated and of a sufficient intelligence to realise that such a policy is morally wrong in the individual and a danger to the State. What may be a sin in the labouring man becomes a crime in the educated brain-worker.

We fear that the medical profession is not wholly free from it. To "ca' canny" cannot be done with piece-work, and most medical work is paid on the piece-work scale. When, however, time-work is taken up by doctors the possibility of this demoralisation creeping into the profession confronts us. We have heard lately of a practitioner who went to work as a specialist under the Ministry of Pensions. The session was for two and a-half hours, during which he was to give an expert opinion upon such cases as were sent to him by the Presidents of the various boards. In the same room was a brother specialist, who had been doing the work for some three months, and within the first half-hour this latter gave advice to the following purpose to the newcomer, "Look here! one piece of advice I would impress upon you, don't work too hard. If you get through many cases in the morning they will only send more cases for you to see in the afternoon."

Amongst our own and other educated sections of the community the cure for such an ill is the prompt recognition that this evil has crept into our life, and the determination of every true man and woman in the profession not to countenance such misconduct. Amongst the labouring class, however, it is more difficult, owing to the fact that the very thing which the working man is now urged not to do has been enforced as a means of relieving unemployment in the past. Whenever bad times arise in the cotton industry arrangements are made for short time in order to avoid dismissal. By making everyone work short, total unemployment is avoided. Now unemployment is the nightmare of the worker's life. He fears it every recurring winter, and fear is fatal to reason. The Premier said, "Until something is done I am afraid it is idle to go to the workers and try to convince them of the fallacy of the doctrine that less work means more employment." Here we have the crux of the problem. A paradox is always difficult of understanding, but when it faces one in the guise of heads I lose and tails you win, it is beyond the reasoning-power of even the most altruistic.

Another point upon which Mr. Lloyd George touched is the plea of Labour which says "We are as human as anybody else, and we do not work well any more than anybody else unless we work with a will." Here again is a factor upon which too much stress cannot be laid. It is the difference between work and drudgery. Work is the greatest happiness that comes to man, but by work we mean an occupation in which a man can be content, and no one can be content unless he can see a return for honest work which will give him a reasonable amount of comfort in life above its bare necessities. If he cannot get this it becomes drudgery and the quality and quantity of the thing done at once deteriorates. We have, therefore, to provide to free every worker from drudgery and to secure that workers of all classes should be always true to their own self-respect and sense of honour.

The views stated above need some slight modification for the present time. The nation is now like a convalescent patient, and we all know that no patient can get well as a result of the doctor's unassisted effort. The patient also must make an effort. The Premier said "There is no recovery without conscious effort on the part of the patient himself. It is a matter of will, and of goodwill," and in saying this shows his grasp of human nature. We can then as a temporary measure ask the worker to put his back into it without any immediate improvement to his own condition if we can guarantee that improvement to him when the nation shall have recovered to its full health.

One of the means to be taken to gain this end must claim the attention of all those who deal with the health of the nation. It is the need to humanise industry. As a part of this the improvement of shops, the proper allocation of time to the character of the work, the mental relaxation of the work, the food of the worker, are all physiological problems which must more and more engage the interest of our profession. Some of us will give all our time to it, and all of us must give an increased proportion.

